# Glutamine stress promotes metastatic potential via hexosamine biosynthetic pathway activity in KRAS/STK11-mutant lung adenocarcinoma

**DOI:** 10.21203/rs.3.rs-10670097/v1

**Published:** 2026-08-27

**Authors:** Shannon Prior, Cole Royer, Logan Sands, Sean Lenahan, Melissa Scheiber, Eyal Amiel, Allison Racela, David Seward, Paula Deming

**Affiliations:** 1 Department of Biomedical and Health Sciences, University of Vermont, Burlington, VT, United States; 2 University of Vermont Cancer Center, University of Vermont, Burlington, VT, United States; 3 Department of Pathology and Laboratory Medicine, University of Vermont, Burlington, VT, United States

## Abstract

Lung cancer is the leading cause of cancer-related deaths worldwide. Loss of STK11 in KRAS-driven lung adenocarcinoma (LUAD) is observed in approximately 15,000 US lung cancer cases annually and drives an aggressive, resistant disease. STK11 regulates many cellular processes, including metabolism, and loss of this tumor suppressor drives a “glutamine addicted” phenotype that is being considered for targeted therapy. However, recent work from our group revealed the activation of pro-oncogenic signaling in KRAS/STK11-mutant LUAD cells upon glutamine deprivation, suggesting the development of an advantageous adaptation. Here, we demonstrate that STK11 loss in KRAS-driven LUAD cells increases glutamine-dependent mitochondrial respiration, which is metabolically rewired upon exogenous glutamine deprivation to enhance the hexosamine biosynthetic pathway (HBP). Furthermore, our results reveal that enhanced HBP flux in STK11 null KRAS-driven LUAD cells promotes a pro-metastatic phenotype characterized by adherent cell detachment, resistance to apoptosis, and 3D spheroid invasion. This study highlights, for the first time, enhanced HBP flux as a protective shunt in response to glutamine deprivation in KRAS/STK11-mutant LUAD. Our results challenge the potential benefit of glutamine deprivation as a therapeutic intervention in this patient population. Future work aims to further elucidate the role of the HBP in promoting metastasis and to determine the mechanism(s) by which STK11 null KRAS-driven LUAD cells upregulate the HBP.

## Introduction

Lung cancer remains the leading cause of cancer-related deaths in the US [1]. Patients with KRAS-mutant lung adenocarcinoma (LUAD) harboring concurrent loss-of-function mutations in STK11 (KRAS/STK11) often present with aggressive disease characterized by increased metastatic spread [2] and resistance to therapy [3]. Advances in genomic medicine have improved cancer treatment by enabling molecular classification and precision therapies tailored to individual tumor profiles. However, these advances have not yet translated into effective treatment options for patients with KRAS/STK11-mutant LUAD, highlighting the need for further research into the molecular underpinnings of this genetic profile.

The tumor suppressor, STK11, acts as a master kinase to regulate signaling networks involved in metabolism, proliferation, survival, and cell migration upon nutrient stress [4]. Loss of STK11 in KRAS-driven LUAD has been shown to result in enhanced glutamine-dependent metabolic rewiring to drive energy homeostasis [5-7]. While cancer therapies aimed at exploiting glutamine addiction represent an attractive therapeutic approach for such vulnerable cancers, it is important to note that deprivation of glutamine can paradoxically induce stress responses that promote tumor progression by selecting for cells capable of metabolic adaptation. Indeed, studies show that glutamine restriction can induce dedifferentiation [8], drug resistance [9], epithelial–mesenchymal transition [10], and nutrient-scavenging behaviors such as micropinocytosis [11-13] - all of which are characteristics of aggressive cancer. Previous work by our group revealed transcriptional upregulation of pro-oncogenic signaling pathways in KRAS/STK11-mutant LUAD cell lines following exogenous glutamine deprivation [14], supporting the existence of an advantageous adaptive response in this LUAD subtype that remains to be elucidated.

In addition to driving glutamine dependency, STK11 loss in KRAS-driven LUAD cells has been shown to promote the flux of glutamine through the hexosamine biosynthetic pathway (HBP) [15]. The HBP is a heavily regulated cytoplasmic metabolic pathway used to generate uridine diphosphate N-acetylglucosamine (UDP-GlcNAc) for glycosylation and O-GlcNAcylation. The plasticity of the HBP enables it to function as a metabolic reserve that can be rapidly upregulated under conditions of nutrient stress or increased demand [16]. Given its adaptive role, the HBP serves as a metabolic hub to drive multiple cancer-promoting pathways during cellular stress [17]. For instance, O-GlcNAcylation, the addition of a single GlcNAc moiety onto serine or threonine residues of target proteins, activates oncogenic transcription factors that increase the metastatic potential of cancer cells [18-20]. Kim et al. demonstrated upregulation of HBP flux in KRAS/STK11-mutant LUAD cells and correlated increased HBP enzyme expression with poor patient prognosis [15]. However, it remains to be established how nutrient stress, specifically glutamine restriction, affects metabolic rewiring in KRAS/STK11-mutant LUAD cells and consequently regulates HBP-dependent processes that may underly tumor progression.

In this study, we demonstrate that depletion of exogenous glutamine induces metabolic rewiring in KRAS/STK11-mutant LUAD cells such that endogenous glutamine is redirected from the TCA cycle towards the HBP. Consistent with reports of metabolic plasticity and glutamine dependency, the results reported here indicate that this adaptive shift promotes a pro-survival phenotype in STK11 null cells that mimics the aggressive nature of nutrient-deprived, metastasizing cancers *in vivo*. Specifically, increased HBP activity promotes key features of metastatic competence such as loss of adhesion, resistance to anoikis, and increased spheroid invasion. These findings support a model in which glutamine-addicted KRAS/STK11-mutant LUAD cells respond to glutamine scarcity, a characteristic feature of the tumor microenvironment, by reallocating intracellular glutamine stores to the HBP thereby coupling metabolic adaptation to the acquisition of a pro-metastatic phenotype.

## Materials and Methods

### Cell culture and treatment

NCI-H2009 (ATCC# CRL-5911) and NCI-H441 (ATCC# HTB-174) cells with (“parental”) and with STK11 knocked-out (ΔSTK11; “STK11 null”) [14] were cultured in RPMI-1640 (Gibco, 11875093) supplemented with 10% fetal bovine serum (FBS) (Corning, 35-011-CV) and L-Glutamine (Gibco, 25030081) (4 mM final concentration; “full RPMI”). The following reagents were used for cell maintenance and treatments: PBS (Gibco, 10010023), 0.25% Trypsin (Gibco, 25200056) and RPMI-1640 without L-Glutamine (Gibco, 21870076). Cells were passaged, maintained in full RPMI and treated at ~80% confluency unless otherwise specified. For glutamine deprivation experiments, cells were washed with 1X PBS at confluency and then either RPMI-1640 without L-Glutamine (“glutamine deficient”) or RPMI-1640 without L-Glutamine supplemented with 2 mM L-Glutamine (“glutamine proficient”) was added for the indicated time(s).

### Seahorse mitochondrial stress test

The Seahorse mitochondrial stress test (MST) was performed as described previously [21]. In brief, cells were seeded in glutamine proficient or deficient RPMI media, as indicated, and allowed to culture for 5 hours before media was swapped to respective 0 mM or 2 mM glutamine-containing Seahorse XF RPMI media (Agilent, 103576-100) supplemented with 11.1 mM glucose (Gibco, A2494001) and 10% FBS. Oligomycin (Thermo Fisher Scientific, J51898MA), fluoro-carbonyl cyanide phenylhydrazone (FCCP; Cayman Chemical, 15218), and rotenone (MP Biochemicals, 150154)/antimycin (Sigma Aldrich, A8674) were used for MST injections. Quantitation was performed on 3 biological replicates (n=3) using an ordinary two-way Anova with Tukey’s multiple comparisons test, with a single pooled variance. Significance was defined as p ≤ 0.05 (95% CI).

### Glutamine metabolic tracing

H2009 cells were incubated in glutamine-free RPMI media supplemented with 10% FBS and 4 mM ^13^C^15^N-glutamine (Cambridge Isotope Laboratories, 285978-14-5) for 4 hours. Glutamine deficient RPMI was then added for 6 or 24 hours, as indicated. Samples were sent to the University of Colorado Anschutz Metabolomics Core where global unlabeled metabolomic analysis along with ^13^C^15^N-glutamine flux analysis was performed via ultra-high performance liquid chromatography-mass spectrometry (n = 3).

### Western and far western blots

Whole cell protein extracts (WCE) were prepared using NP40 lysis buffer (25 mM Tris pH 7.2, 137 mM NaCl, 25 mM NaF, 10 mM Na pyrophosphate, 10% glycerol and 1% IGEPAL; pH 7.4) supplemented with 1X protease inhibitor (Thermo Fisher, 78441) and 20 μM Thiamet-G (TMG) (ApexBio, B2048). Extracts were centrifuged at 14,000 x g for 10 min at 4°C and protein concentrations were then determined using the Pierce BCA Protein Assay Kit (Thermo Fisher, 23227). Twenty μg of WCE were subjected to SDS-PAGE electrophoresis using 8% acrylamide (Protogel, EC-890) at 200 V for 1 hour. Gels were then transferred to nitrocellulose membranes (BioRad, 1704270), blocked in Tris-buffered saline with 0.1 % Tween 20 (Thermo Fisher, P7949) (TBST) containing 1% bovine serum albumin (BSA) (Thermo Fisher, 9048-46-8) for 1 hour at room temperature (western blot) or overnight at 4°C (far western blot). Sequential incubation with target-specific primary antibodies (overnight at 4°C) or biotin-tagged wheat germ agglutinin (WGA) lectin (Vector Labs, B-1025-5; 2 hours at room temperature), followed by appropriate horseradish peroxidase (HRP)-conjugated secondary antibodies (western blot) or streptavidin (far western blot) was then performed (Table 1). Resulting bands were visualized using a chemiluminescence substrate (Thermo Fisher, 1863094). Full, uncropped images of all blots are provided in Supplementary information. Densitometry was performed using ImageJ on 3 biological replicates (n=3) and significance was determined using an ordinary two-way Anova with Tukey’s multiple comparisons test, with a single pooled variance. Significance was defined as p ≤ 0.05 (95% CI).

### Hexosaminidase treatment

Cells lysates were normalized to 1 mg/mL concentration and 40 μL β-*N*-acetylhexosaminidasef (New England Biolabs, P0721S) was added per 1 mg of protein. Mixtures of protein lysates and hexosaminidase were incubated at 37°C for 1 hour and then prepped for SDS-PAGE and subsequent western and far western blot analysis as described above. Corresponding untreated samples were used for the analysis of the time course of glutamine deprivation.

### FR054 treatment

FR054 (MedChemExpress, 35954-65-5) was solubilized in DMSO (Fisher Scientific, 191418) to a stock concentration of 10 mM and treated at concentrations indicated. For detachment and apoptosis assays cells were pretreated for 3 hours with 50 μM FR054 prior to continued treatment in glutamine proficient or deficient media. For 3D invasion experiments, in accordance with previous studies [22], an increased dose of 250 μM FR054 was used.

### Quantification of detached cells

Detached cells were isolated via centrifugation at 135 x rcf for 5 min at 4°C. Cell pellets were resuspended in 100 μL respective media and live/dead cells were quantified using Trypan Blue (Gibco, 15250061). Quantitation was performed on 3 biological replicates (n=3) using an ordinary two-way Anova with Tukey’s multiple comparisons test, with a single pooled variance. Significance was defined as p ≤ 0.05 (95% CI).

### Annexin V and propidium iodide (PI) apoptosis assay

Detached cells were isolated via centrifugation at 135 x rcf for 5 min at 4°C. Cell pellets were resuspended in 100 uL AnnexinV Binding Buffer (BioLegend, 422201) and stained with AnnexinV and propidium iodide at ratios of 0.35:100 and 10:100. Flow cytometry was performed using the BD LSRII (BD Biosciences) and data acquisition and analysis were performed with assistance from the UVM Flow Cytometry and Small Particles Detection Facility using BD FACSDiva v8 software (BD Biosciences). Cells were gated on single-stain controls for each experiment, with cells staining negative for both annexin V and PI deemed “non-apoptotic” while “apoptotic” cells were cumulatively referred to as annexin V positive. Quantitation was performed on 3 biological replicates (n=3) using an ordinary two-way Anova with Tukey’s multiple comparisons test, with a single pooled variance. Significance was defined as p ≤ 0.05 (95% CI).

### Quantification of reestablished cells post-detachment

Fifty-thousand detached trypan-negative cells/condition into wells of a 24-well plate and cultured for 7 days prior to fixation with 4% paraformaldehyde and staining with 1X DAPI. Re-adherent cells were quantified on the Lionheart FX Live Cell Imager (BioTek) using 9x6 4x PCH and DAPI objectives. Images were taken using the Gen5 BioTek software (Gen5Image + 3.06) and DAPI images were stitched. Utilizing the cell count feature, objects greater than 5 μm were quantified to produce cell counts per well. Quantitation was performed on 3 biological replicates (n=3) using an ordinary one or two-way Anova with Tukey’s multiple comparisons test, with a single pooled variance. Significance was defined as p ≤ 0.05 (95% CI).

### Spheroid 3D invasion

Spheroids were generated using a modified hanging drop method [23], after which they were washed and resuspended in Matrigel (Corning, 356231) diluted to 5 mg/mL in glutamine proficient or deficient media. For time-point imaging of invasion, spheroids were cultured for 48 hours and imaged using a Laxco, LMI6-FL12 inverted microscope. Single disseminated cell counts from 3 biological replicates of spheroids were obtained using Fiji (magej.net/software/fiji/). For live-cell imaging of invasion, spheroids were cultured at 37°C and 5% CO_2_ for 48 hours (H2009) or 72 hours (H441) and imaged using the LIONHEART FX (BioTek, Winooski, VT) every 30 min. Images were stitched and converted to videos using the Gen5 Software (Gen5Image+ 3.06). Quantitation was performed on pooled spheroid invasion from 3 biological replicates (n=3) using an ordinary one or two-way Anova with Tukey’s multiple comparisons test, with a single pooled variance. Significance was defined as p ≤ 0.05 (95% CI).

## Results

### Loss of STK11 drives increased glutamine-dependent mitochondrial respiration in KRAS-driven LUAD cells

**Figure 1.**
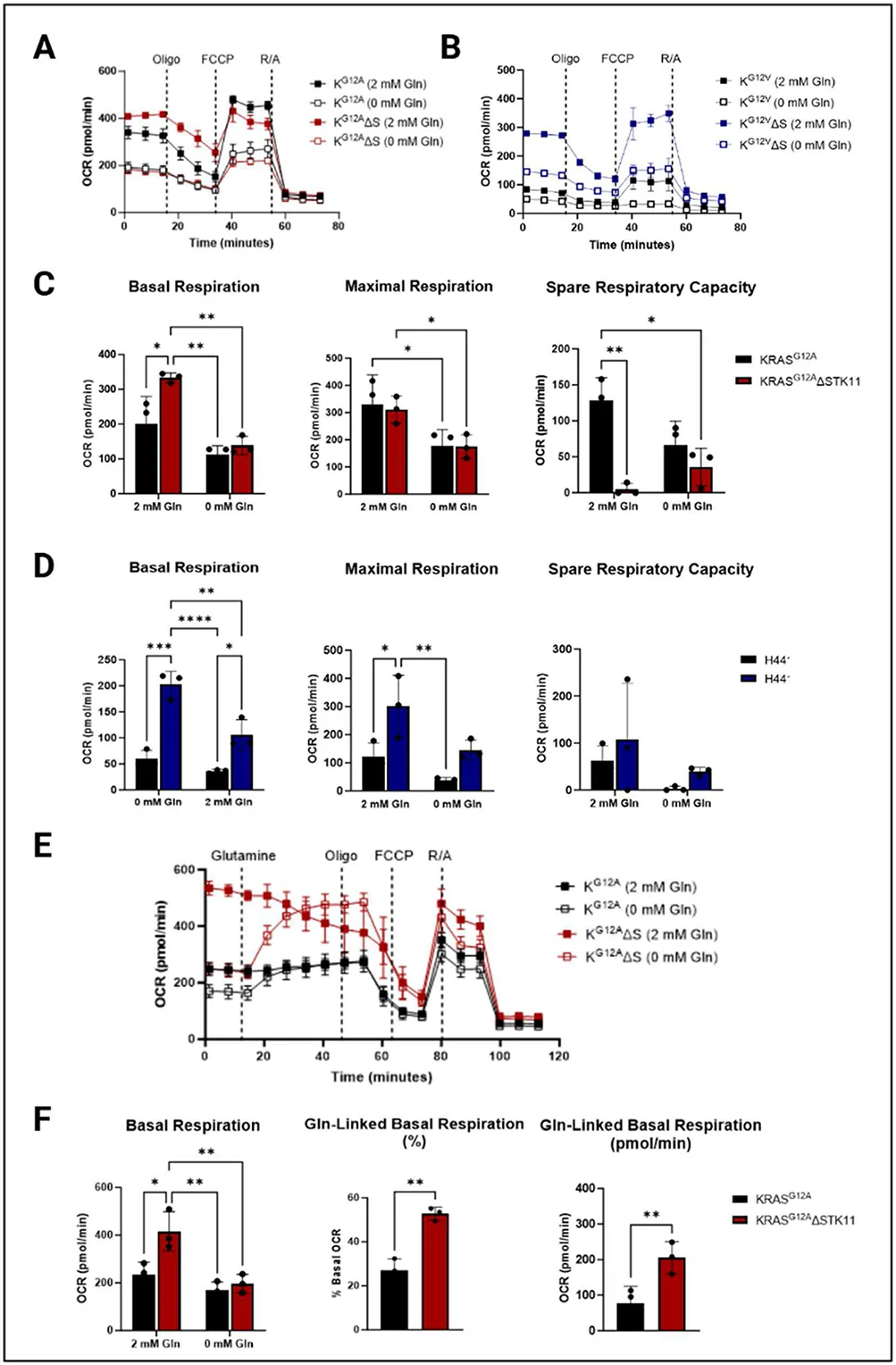
STK11 loss drives enhanced glutamine-dependent mitochondrial respiration in KRAS-driven LUAD cells. **(A)** Representative oxygen consumption rate (OCR) traces of a Seahorse Mitochondrial Stress Test (MST) in NCI-H2009 KRAS^G12A^ and KRAS^G12A^ΔSTK11 cells treated with either 0 mM or 2 mM glutamine. Cells were seeded in respective glutamine deficient or replete media approximately 5 hours prior to and during the MST. **(B)** Representative OCR traces of a Seahorse MST in NCI-H441 KRAS^G12V^ and KRAS^G12V^ΔSTK11 cells treated with either 0 mM or 2 mM glutamine. Cells were seeded in respective glutamine deficient or replete media approximately 5 hours prior to and during the MST. **(C)** Quantification of H2009 KRAS^G12A^ and KRAS^G12A^ΔSTK11 primary MST parameters (basal respiration, maximal respiration and spare respiratory capacity (SRC)) in 0 mM and 2 mM glutamine conditions (n = 3; two-way ANOVA with Tukey’s multiple comparisons test). **(D)** Quantification of H441 KRAS^G12D^ and KRAS^G12D^ΔSTK11 primary MST parameters (basal respiration, maximal respiration and spare respiratory capacity (SRC)) in 0 mM and 2 mM glutamine conditions (n = 3; two-way ANOVA with Tukey’s multiple comparisons test). **(E)** Representative traces of a modified Seahorse MST in which cells were seeded in respective glutamine deficient or replete media approximately 5 hours prior to the MST and then treated with an injection of 2 mM glutamine and allowed to equilibrate before the MST injections. **(F)** Quantification of H2009 KRAS^G12A^ and KRAS^G12A^ΔSTK11 modified Seahorse MST parameters – BR, % glutamine-linked BR (defined as ((+gln BR – −gln BR)/+gln BR) * 100)), and glutamine-linked BR as OCR (defined as (−gln OCR post-glutamine injection – −gln BR)) (n = 3; two-way ANOVA with Tukey’s multiple comparisons test). Oligo = oligomycin, R/A = rotenone/antimycin. * p < 0.05, ** p < 0.01, standard deviation (SD) shown.

Given the heightened dependency on glutamine metabolism in KRAS/STK11-mutant LUAD, we used the Seahorse Mitochondrial Stress Test (MST) [21] to assess the contribution of glutamine in driving mitochondrial respiration upon STK11 loss in KRAS-driven LUAD [24]. As shown in Figure 1, both H2009 (Fig. 1A and Fig. 1C) and H441 (Fig. 1B and Fig. 1D) STK11 null cells displayed elevated basal oxygen consumption rates (OCR) compared to parental lines. Interestingly, in H2009 cells, STK11 null cells almost entirely lacked spare respiratory capacity (SRC) in nutrient replete conditions, indicating a “maxed out” bioenergetic status and inability to respond to metabolic stress at baseline [21]. Upon glutamine deprivation, while parental cells had reduced SRC, H2009 STK11 null cells demonstrated an increase (Fig. 1C), suggesting such nutrient stress results in a shift from full capacity to a state of energy conservation. A similar result indicative of metabolic efficiency was observed in the H441 line in which STK11 null cells maintained an SRC following the depletion of glutamine, while the SRC of parental cells was entirely abolished (Fig. 1D). Of note, extracellular acidification rates (ECAR) of both H2009 cell lines were comparable throughout the MST, indicating sustained glycolysis following glutamine deprivation (Fig. S1A-B). Additional MST parameters are reported in Supplemental Figure S1.

We next performed a modified MST to confirm the contribution of glutamine in driving the elevated basal respiration observed in STK11 null cells. Glutamine-deprived H2009 cells received an initial injection of glutamine and OCR levels were allowed to reach a steady state prior to MST injections (Fig. 1E). As expected, the glutamine-driven basal respiration of H2009 STK11 null cells was approximately two-fold that of the H2009 cells (Fig. 1F).

Lastly, to elucidate the timing at which STK11 null cells switch from a hypermetabolic to a stress-primed state upon glutamine stress, we performed a time course of deprivation prior to the MST in our H2009 model. Three hours of glutamine deprivation reduced the basal OCR of H2009 STK11 null cells to levels similar to parental cells in glutamine-rich media, with only a slight additional reduction observed at the 5-hour deprivation time point (Fig. S1E). Given these results, subsequent experiments were focused on the early hours (1-3 hours) of glutamine deprivation.

Taken together, these results reveal that KRAS-driven LUAD cells lacking functional STK11 have greater mitochondrial respiration in nutrient rich conditions, largely due to enhanced glutamine utilization. Upon early hours of glutamine deprivation, however, there appears to be a shunt away from mitochondrial respiration. We hypothesize that this could be an advantageous adaptation upon STK11 loss, preserving the ability of these cells to survive under such metabolic stress.

### Glutamine deprivation rewires endogenous glutamine flux from the TCA cycle to the HBP in KRAS/STK11-mutant LUAD cells

**Figure 2.**
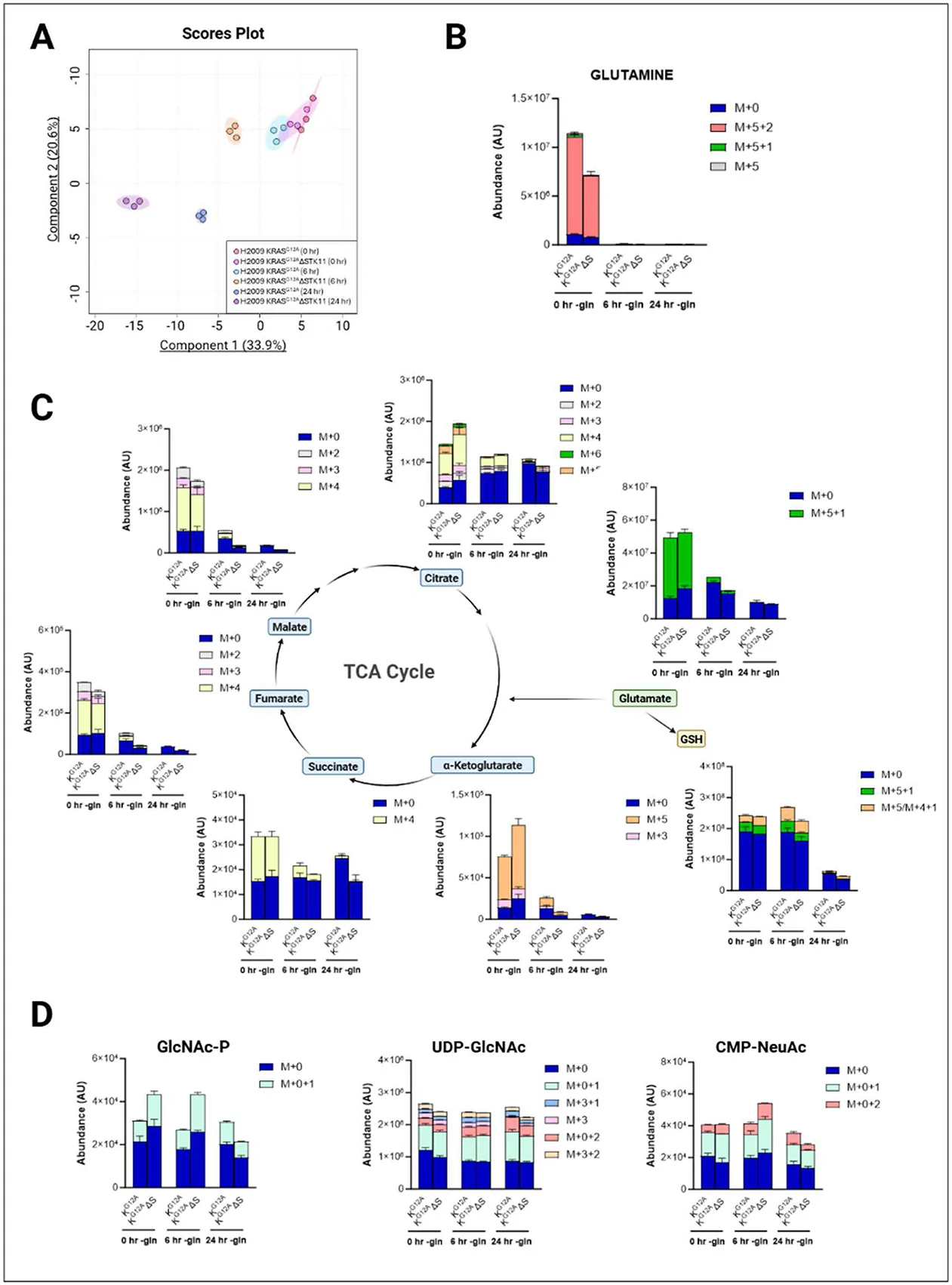
Exogenous glutamine deprivation promotes a shunt of endogenous glutamine from the TCA cycle to the HBP in KRAS/STK11-mutant LUAD cells. **(A)** Principal component analysis (PCA) plot demonstrating variance of NCI-H2009 KRAS^G12A^ and KRAS^G12A^ΔSTK11 cells following a modified pulse-chase experiment in which cells were pulsed with mM ^13^C^15^N-glutamine for 4 hours which was then chased following exogenous glutamine deprivation for either 0 (i.e. immediately post-pulse), 6 or 24 hours. Global unlabeled metabolic and stable ^13^C^15^N-glutamine flux analysis were performed via UHPLC-MS/MS at the University of Colorado Anschutz Metabolomics Core (n = 3). **(B)** Abundance of glutamine (both labeled and unlabeled) in NCI-H2009 KRAS^G12A^ (K^G12A^) and KRAS^G12A^ΔSTK11 (K^G12A^ΔS) cells subjected to the modified pulse-chase experiment. **(C)** Abundance (both labeled and unlabeled) of glutamate, glutathione and TCA intermediates, α-ketoglutarate, succinate, fumarate, malate and citrate in NCI-H2009 KRAS^G12A^ (K^G12A^) and KRAS^G12A^ΔSTK11 (K^G12A^ΔS) cells subjected to the modified pulse-chase experiment. **(D)** Abundance (both labeled and unlabeled) of the HBP intermediate, GlcNAc-phosphate (GlcNAc-P), the HBP product, UDP-GlcNAc, and the downstream HBP metabolite, CMP-sialic acid (CMP-NeuAc) in NCI-H2009 KRAS^G12A^ (K^G12A^) and KRAS^G12A^ΔSTK11 (K^G12A^ΔS) cells subjected to the modified pulse-chase experiment.

Given the unexpected finding that exogenous glutamine deprivation, a characteristic of the tumor microenvironment [25-27], reduced SRC in STK11 proficient cells but led to increased or sustained SRC in STK11 null cells, we decided to next examine metabolic flux. Heavy-labeled glutamine (^13^C^15^N-glutamine) was added to H2009 cell culture media for 4 hours, followed by a swap to glutamine-deficient media for 0, 6 and 24 hours. As shown in the principal component analysis (PCA) in Figure 2A, parental and STK11 null cells clustered similarly immediately post-pulse of ^13^C^15^N-glutamine, however there was a distinct difference at 6 hours of deprivation that was exacerbated by 24 hours. Of note, glutamine levels in STK11 null cells were reduced at baseline compared to those in parental cells (Fig. 2B), indicating either decreased uptake of labeled glutamine or increased utilization - the latter of which many studies support [5-7]. By 6 hours of exogenous glutamine deprivation, and maintained at 24 hours, levels of unlabeled and labeled glutamine were abolished in both H2009 cell lines, revealing the rapid utilization of intracellular glutamine upon extracellular scarcity. In correspondence with our Seahorse data, deprivation of glutamine for 6 hours resulted in decreased flux to all TCA cycle intermediates measured in both H2009 cell lines (Fig. 2C). The top 50 unlabeled metabolites from this modified pulse chase experiment are reported in Supplemental Figure S2.

To investigate whether there is a metabolic “sink” to which intracellular glutamine is shunted upon extracellular glutamine stress, additional heavy labeled glutamine-derived metabolites were examined. Interestingly, we observed maintenance of hexosamine biosynthetic pathway (HBP) metabolites, GlcNAc-phosphate (GlcNAc-P) and UDP-GlcNAc, in H2009 STK11 null cells. Furthermore, CMP-sialic acid (CMP-NeuAc), a downstream activated metabolite of the HBP, was elevated in STK11 null cells following 6 hours of glutamine deprivation (Fig. 2D). Given that O-GlcNAcylation [28] and increased sialylation [29] have been associated with aggressive, pro-metastatic characteristics of cancer cells, this pathway piqued our interest as a potential protective metabolic shunt in KRAS-driven LUAD cells lacking STK11.

**Figure 3.**
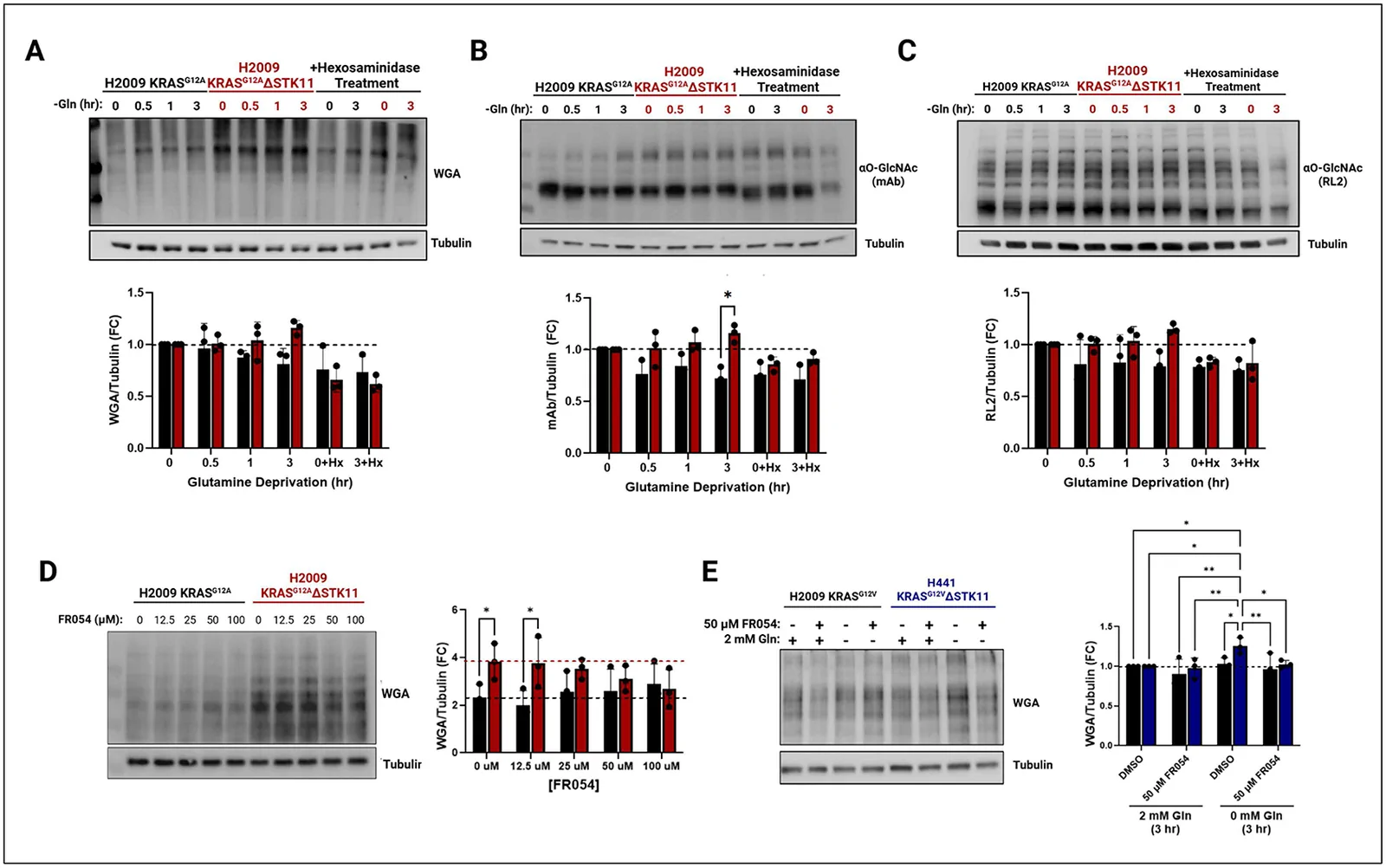
Western and far western blot analyses confirm elevated HBP activity upon glutamine deprivation in KRAS/STK11-mutant LUAD cells. **(A)** Far western blot (wheat germ agglutinin; WGA) and western blot analysis (α-O-GlcNAc antibodies, **(B)** mAB and **(C)** RL2) of a glutamine deprivation time course ranging from 0 – 3 hours in NCI-H2009 KRAS^G12A^ (black) and KRAS^G12A^ΔSTK11 (red) cells. For both control (i.e. 0 hours glutamine deprivation) and 3-hour time points, a hexosaminidase treatment was included to demonstrate specificity of each probe. Densitometry is displayed as fold-change (FC) of probe/tubulin loading control for each condition relative to the respective cell line 0-hour control (n = 3; two-way ANOVA with Tukey’s multiple comparisons test). * p < 0.05, standard deviation (SD) shown. **(D)** Representative far western blot (WGA) and quantification showing the dose response following 24 hours of treatment with 0 μM to 100 μM FR054 in NCI-H2009 KRAS^G12A^ and KRAS^G12A^ΔSTK11 cells. Densitometry is displayed as fold-change (FC) of WGA/tubulin loading control for each condition, and dashed lines are indicating average basal (i.e. 0 μM FR054) signal (n = 3; two-way ANOVA with Tukey’s multiple comparisons test). **(E)** Representative far western blot (WGA) and quantification of NCI-H441 KRAS^G12V^ and KRAS^G12V^ΔSTK11 cells cultured for 3 hours in either 2 mM or 0 mM glutamine and concurrent DMSO or 50 μM FR054, as indicated. Densitometry is displayed as fold-change (FC) of probe/tubulin for each condition relative to their respective 2 mM glutamine DMSO control (n = 3; two-way ANOVA with Tukey’s multiple comparisons test).

To confirm elevated flux through the HBP upon early hours of glutamine deprivation, we used a combination of western blot and far western blot approaches. The lectin, wheat germ agglutinin (WGA), was used to measure both global sialylation and O-GlcNAcylation while two different antibodies (multi mAb and RL2; Table 1) were used to specifically measure global O-GlcNAcylation. As shown in Figure 3, and in support of the metabolic flux analysis, both lectin (Fig. 3A) and antibodies (Fig. 3B and 3C) revealed a similar trend in signal following glutamine deprivation. Specifically, while signals across all probes diminished over early hours of glutamine deprivation in H2009 parental cells, there was a consistent increase in signals in STK11 null cells that was most pronounced at the 3-hour deprivation time point. We verified the results of all probes with hexosaminidase treatment, which cleaves O-GlcNAc residues from proteins thus confirming the specificity of O-GlcNAc signal [30] (Fig. 3A-C).

The selective small-molecule inhibitor, FR054, targets the intermediary HBP enzyme phosphoglucomutase 3 (PGM3) and has demonstrated efficacy in both breast cancer [31] and KRAS/STK11-mutant lung cancer cells [32], and thus was used to inhibit HBP activity in our cell model system. As shown in Figure 3D, 50 μM FR054 was sufficient to reduce sialylation and O-GlcNAcylation levels in H2009 STK11 null cells while the parental line remained relatively unaltered. Validation of HBP induction following 3 hours of glutamine deprivation along with sensitivity to 50 μM FR054 was performed in H441 cells using the WGA lectin-based far western approach (Fig. 3E). Together, these results highlight novel metabolic rewiring during glutamine scarcity and a potential shift to a pro-metastatic state in KRAS-driven LUAD cells that lack STK11.

### HBP activity in KRAS/STK11-mutant LUAD cells promotes cell detachment and apoptotic resistance upon glutamine deprivation

**Figure 4.**
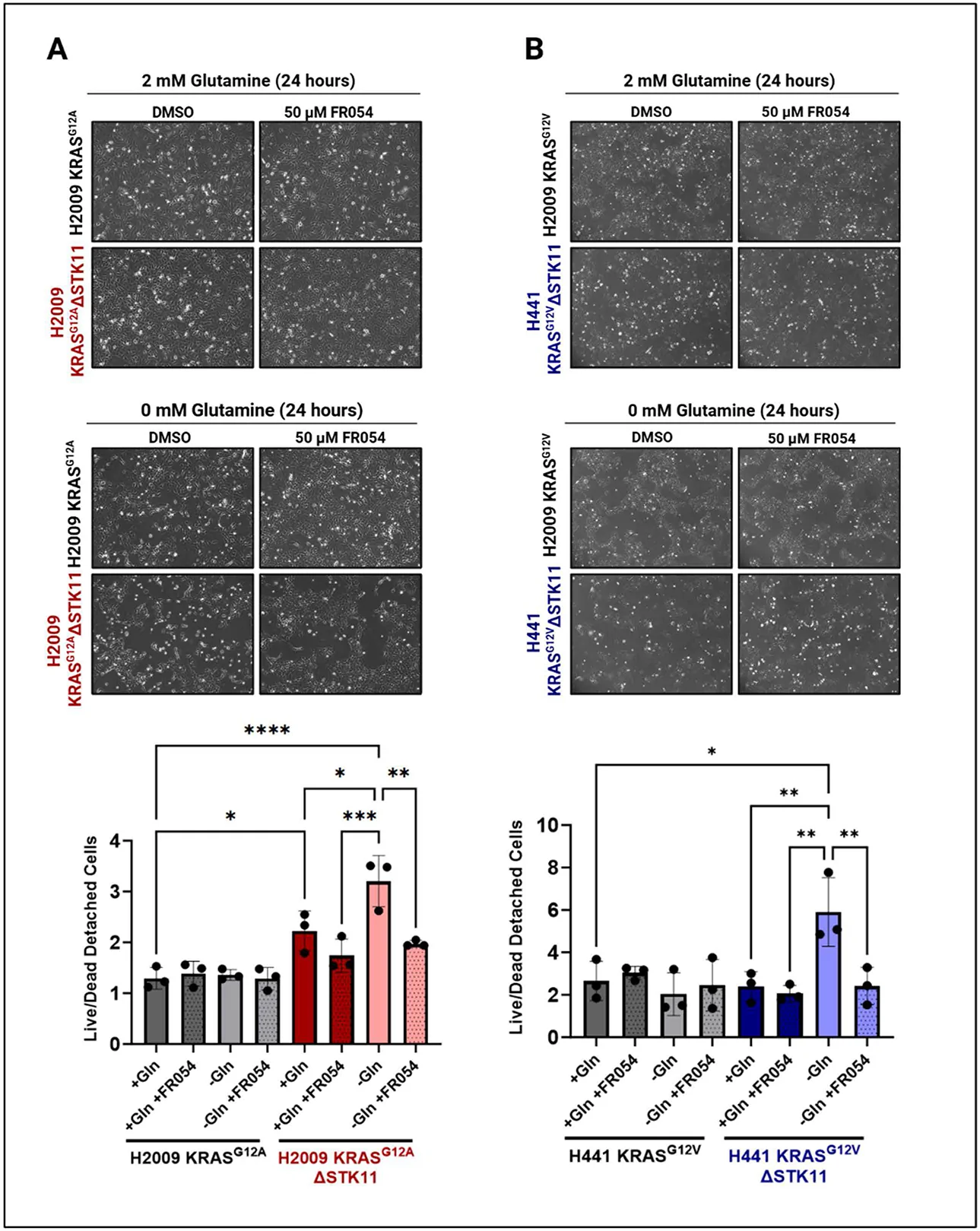
HBP activity induced upon glutamine deprivation promotes detachment and survival in KRAS/STK11-mutant LUAD cells. **(A)** Representative 10X figures and quantification of NCI-H2009 KRAS^G12A^ and KRAS^G12A^ΔSTK11 cells, and **(B)** NCI-H441 KRAS^G12V^ and KRAS^G12V^ΔSTK11 cells subjected to 24 hr of culture in 0 or 2 mM glutamine and 0 (i.e. DMSO vehicle) or 50 μM FR054, as indicated. Quantification was performed using Trypan Blue stain and analysis is presented as ratios of Trypan Blue exclusive/inclusive (i.e. “live/dead”) cells for each cell line (n = 3; one-way ANOVA with Tukey’s multiple comparisons test). * p < 0.05, standard deviation (SD) shown.

While conducting glutamine deprivation experiments, a striking observation was made - upon 24 hours of extracellular glutamine starvation, STK11 null cells detached at a much greater level than parental cells and, surprisingly, appeared to be viable as evidenced by Trypan Blue staining (Fig. 4 and S3). Survival of cells following detachment from the extracellular matrix is widely considered the initiating step in the metastatic cascade as it enables dissemination of tumor cells from their primary site [33]. Our finding that glutamine deprivation increases HBP products in STK11 null cells, coupled with evidence that HBP-mediated surface sialylation and O-GlcNAcylation regulate cell adhesion and motility [34], led us to test whether enhanced HBP activity mediates the increased cell detachment phenotype observed in STK11 null cells under glutamine-deprived conditions.

In nutrient replete conditions, H2009 (Fig. 4A) and H441 (Fig. 4B) STK11 null cells displayed a greater ratio of Trypan Blue exclusive (i.e. live) /inclusive (i.e. dead) detached cells as compared to parental cells. However, unlike parental cells which maintained comparable levels of live detached cells under nutrient-replete and glutamine-deprived conditions, STK11 null cells exhibited a significant increase in live cell detachment and thus live/dead cell ratio in response to glutamine deprivation. Treatment with FR054 significantly reduced the number of live detached STK11 null cells under both conditions while leaving parental cells unaltered, indicating the HBP does indeed play a role in driving the detachment phenotype of KRAS/STK11-mutant LUAD cells.

**Figure 5.**
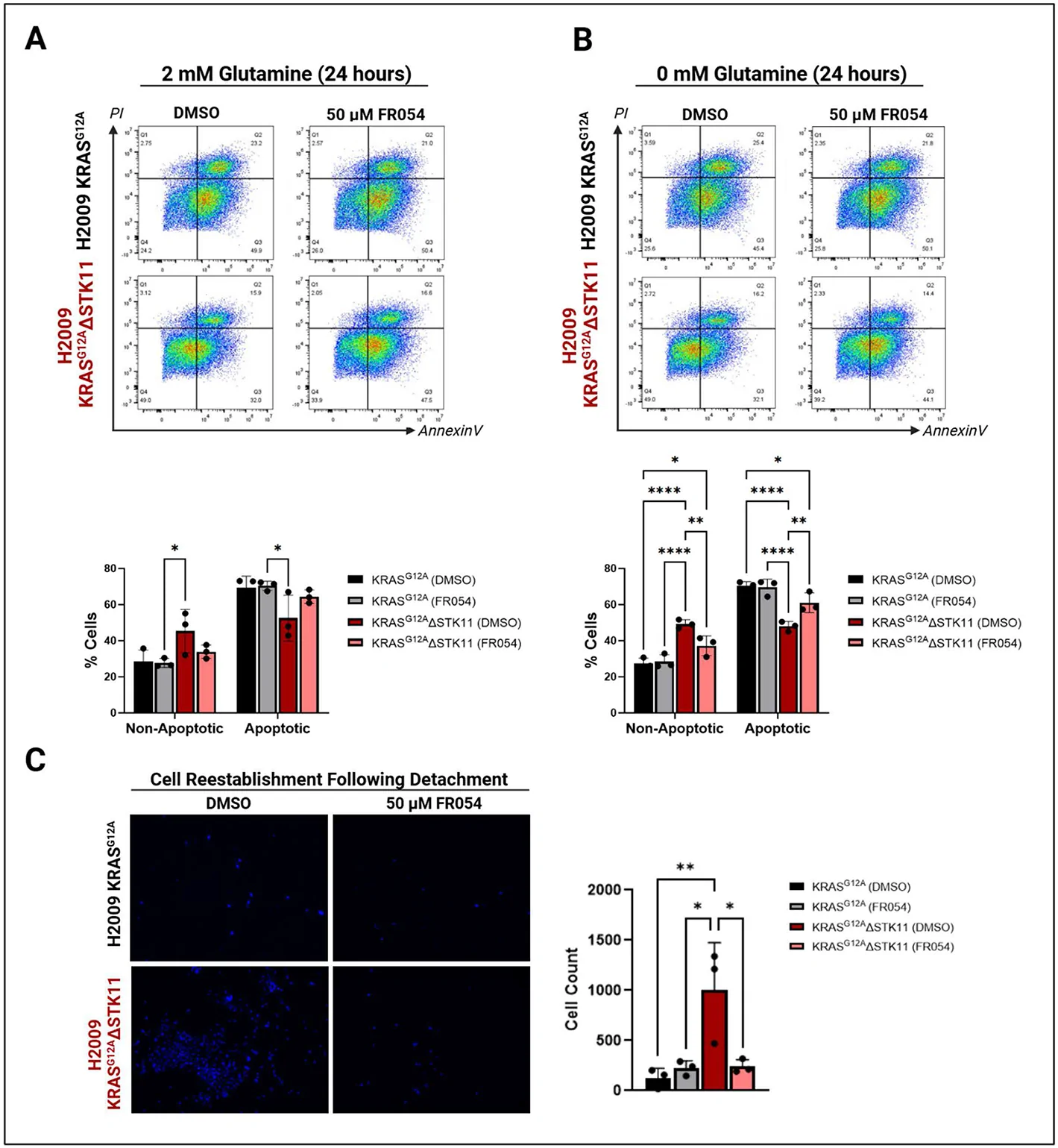
HBP activity induced upon glutamine deprivation promotes apoptotic resistance in detached KRAS/STK11-mutant LUAD cells. Representative flow cytometry plots of detached NCI-H2009 KRAS^G12A^ and KRAS^G12A^ΔSTK11 cells following 24 hours of culture in **(A)** 2 mM or **(B)** 0 mM glutamine-containing media with 0 μM or 50 μM FR054, as indicated. Cells were stained with annexin V and PI for assessment of apoptosis and gated on single stain controls. Data is displayed as percent of entire detached cell population. Non-apoptotic, annexin V-negative cells are gated in quadrant 4 ("non-apoptotic"), while annexin V-positive cells, both alive and dead, are gated in quadrants 3 and 4, respectively (cumulatively referred to as "apoptotic"). Quantification of "non-apoptotic" and "apoptotic" cell populations was performed (n = 3 biological replicates; two-way Anova with Tukey's multiple comparisons test). **(C)** Representative images and quantification of DAPI stained NCI-H2009 KRAS^G12A^ and KRAS^G12A^ΔSTK11 cells following detachment induced by 24 hours of glutamine deprivation and 0 μM or 50 μM FR054, as indicated, and subsequent culture in nutrient replete media for 7 days (re-seeded at 50,000 trypan exclusive detached cells/well) (n = 3; unpaired t-test, two-tailed). * p < 0.05, ** p < 0.01, *** p < 0.001, **** p < 0.0001, standard deviation (SD) shown.

To further investigate the viability of STK11 null cells upon glutamine deprivation-induced detachment, annexin V and propidium iodide staining was performed to measure apoptotic cells, as described previously [35]. Similar to nutrient replete conditions (Fig. 5A), 24 hours of glutamine starvation (Fig. 5B) resulted in significantly more apoptotic detached H2009 parental cells (those staining positive for annexin V and/or propidium iodide) as compared to STK11 null cells. In line with its effect on cell detachment, treatment with FR054 had no effect on the number of apoptotic or non-apoptotic parental cells in either condition. In contrast, STK11 null cells that detached upon glutamine deprivation were significantly affected by FR054 treatment which increased the number of apoptotic cells (Fig. 5B).

Since metastatic cells must survive detachment and subsequently colonize distant tissues, we next investigated whether H2009 cells detached under glutamine-deprived conditions retained the ability to reattach and proliferate when returned to nutrient-replete media. As shown in Figure 5C, detached H2009 parental cells exhibited little to no capacity to re-adhere and survive when replated in nutrient-replete conditions. Detached STK11 null cells, however, demonstrated a significantly greater capacity to reestablish growth – a phenomenon which was markedly reduced upon inhibition of the HBP (Fig. 5C). Overall, these results suggest that enhanced HBP flux in KRAS/STK11-mutant LUAD cells promotes cell detachment, apoptotic resistance, and survival upon glutamine deprivation - all of which are key features of metastasis.

### Inhibition of the HBP blunts invasive phenotypes of KRAS/STK11-mutant LUAD spheroids induced upon glutamine deprivation

**Figure 6.**
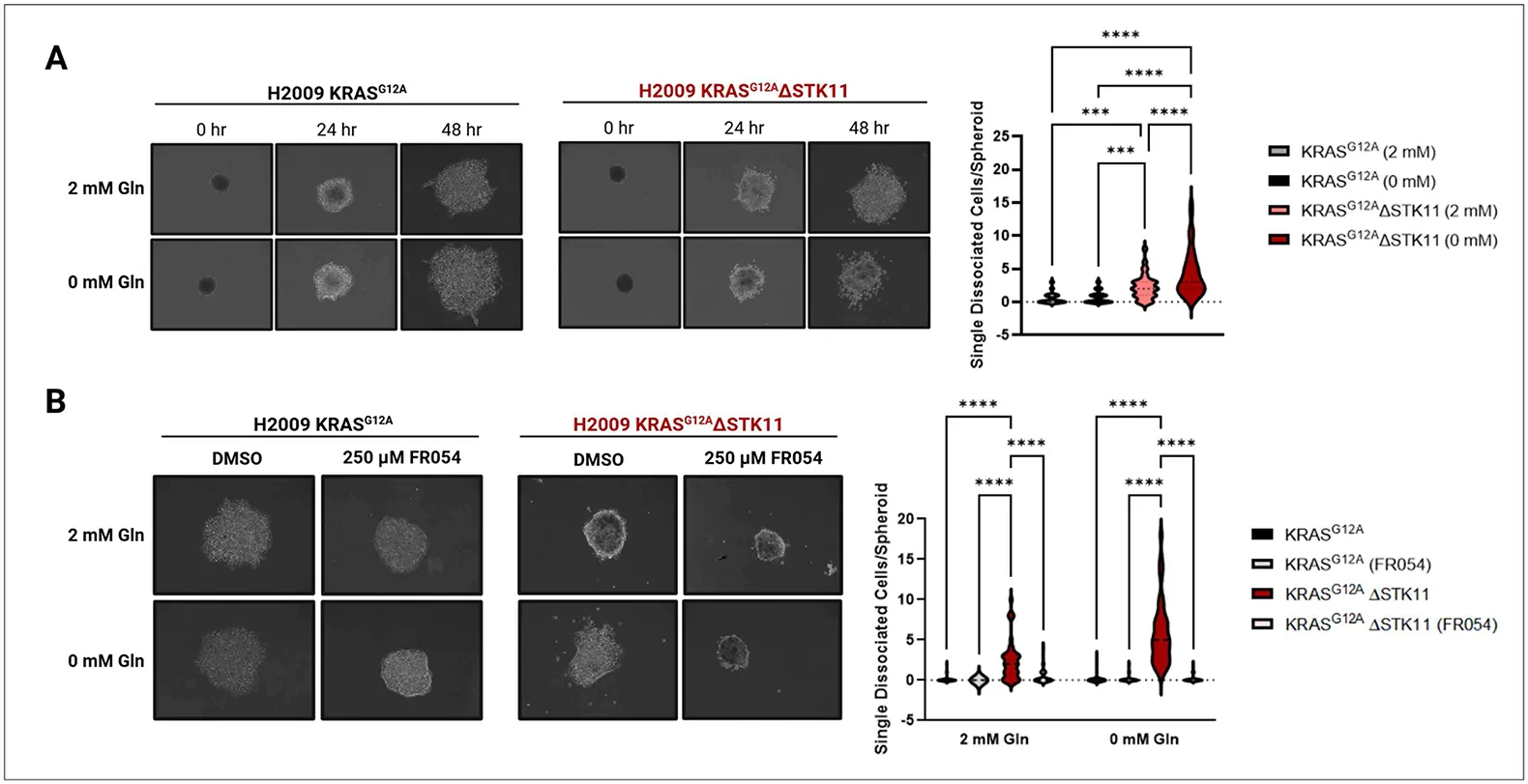
H2009 KRAS/STK11-mutant LUAD spheroids display enhanced single cell invasion upon glutamine deprivation that is abolished with FR054 treatment. **(A)** Representative images of NCI-H2009 KRAS^G12A^ and KRAS^G12A^ΔSTK11 spheroid invasion after 0 hours, 24 hours and 48 hours of culture in glutamine-replete (i.e. 2 mM) and glutamine-deficient (i.e. 0 mM) conditions. Quantification of single cell invasion at 48 hours was performed, with cells that were no longer touching the main spheroid core nor other invading cells at the time of imaging considered as “dissociated cells” (n = 3; varying technical replicates per biological experiment for a total of 40 KRAS^G12A^ (2 mM), 53 KRAS^G12A^ (0 mM), 53 KRAS^G12A^ΔSTK11 (2 mM) and 53 KRAS^G12A^ΔSTK11 (0 mM) spheroids analyzed. One-way ANOVA with Tukey’s multiple comparisons test). **(B)** Representative images of NCI-H2009 KRAS^G12A^ and KRAS^G12A^ΔSTK11 spheroid invasion at 48 hours following culture in glutamine-deficient (i.e. 0 mM) conditions with concurrent 0 μM (i.e. DMSO vehicle) or 250 μM FR054 treatment, as indicated. Quantification of single “dissociated cells” was performed (n = 3; varying technical replicates per biological experiment for a total of 37 KRAS^G12A^ (2 mM + DMSO), 24 KRAS^G12A^ (2 mM + FR054), 32 KRAS^G12A^ (0 mM + DMSO), 32 KRAS^G12A^ (0 mM + FR054), 39 KRAS^G12A^ΔSTK11 (2 mM + DMSO), 35 KRAS^G12A^ΔSTK11 (2 mM + FR054) 38 KRAS^G12A^ΔSTK11 (0 mM + DMSO) and 41 KRAS^G12A^ΔSTK11 (0 mM + FR054) spheroids analyzed. Two-way ANOVA with Tukey’s multiple comparisons test). * p < 0.05, **** p < 0.0001, standard deviation (SD) shown.

Acquisition of an invasive phenotype, characterized by the ability to degrade and traverse the extracellular matrix, is another defining feature of metastatic progression. To evaluate invasive behavior in a physiologically relevant microenvironment, we performed 3D spheroid invasion assays which measure the ability of tumor cells to invade the surrounding extracellular matrix. Using this model, we determined whether HBP activity contributes to the enhanced invasion of STK11 null cells under glutamine-deprived conditions. As shown in Figure 6 and in Supplemental Videos, parental H2009 spheroids maintained uniform, collective invasion for 48 hours regardless of culture in 2 mM versus 0 mM glutamine. In contrast, H2009 STK11 null spheroids displayed a striking single cell invasive phenotype that was significantly enhanced upon glutamine deprivation – an established feature of aggressive, metastasizing cancers [36]. HBP inhibition via FR054 treatment almost entirely abolished the aberrant, single-invasion phenotype of STK11 null cells under glutamine proficient and deficient conditions (Fig. 6A). In contrast, however, FR054 treatment had minimal effect on H2009 parental spheroid invasion regardless of nutrient status, highlighting the significance of STK11 loss in driving such metabolic rewiring. The effect of HBP inhibition was corroborated in H441 spheroids, in which FR054 treatment prevented STK11 null spheroid invasion upon glutamine deprivation while having minimal impact on parental spheroids (Fig. S4 and Supplemental Videos). Interestingly, H441 STK11 null spheroids displayed more of a membrane blebbing phenotype under conditions of glutamine stress rather than single cell dissemination characteristic of H2009 STK11 null spheroids. This distinction suggests that H441 cells may employ an alternative mode of motility in which cell-cell contacts are maintained, consistent with actomyosin-driven membrane blebbing during collective invasion – a migratory phenotype associated with enhanced invasive capacity and metastatic plasticity in multiple cancer models [37]. Taken together, these results support the notion that glutamine scarcity induces increased aggressive, pro-metastatic features in KRAS/STK11-mutant LUAD cells, largely driven by HBP activity.

## Discussion

Metastasis remains the leading cause of cancer-related mortality, accounting for approximately 90% of all patient deaths [38]. Progression from a confined primary tumor to metastatic spread requires tumor cells to adapt to multiple stresses, including nutrient deprivation, loss of matrix attachment, and oxidative stress [33]. KRAS/STK11 mutant LUAD is characterized by aggressive metastatic behavior and profound metabolic rewiring, thus understanding how these tumors adapt to nutrient stress is critical. Here, we demonstrate that glutamine deprivation rapidly redirects intracellular glutamine away from the TCA cycle toward the hexosamine biosynthetic pathway (HBP). This shunt promotes O-GlcNAcylation and sialylation together with increased cell detachment, anoikis resistance, the ability to re-establish growth following detachment, and three-dimensional invasion. Collectively, these findings identify the HBP as an adaptive metabolic program that links glutamine stress to metastatic features.

Although STK11 loss creates a previously recognized dependence on glutamine metabolism in lung cancer, therapeutic strategies targeting glutamine utilization have shown limited clinical success. Recent work has proposed that glutamine stress follows a biphasic hormetic response, in which moderate nutrient stress activates adaptive programs that enhance tumor aggressiveness before more severe stress becomes growth inhibitory or cytotoxic [39] – a model which our findings strongly support. Rather than undergoing metabolic collapse following glutamine deprivation, STK11 null cells rapidly entered an adaptive state characterized by reduced glutamine-derived TCA cycle activity while preserving glutamine flux through the HBP. This metabolic shift coincided with the acquisition of multiple pro-metastatic phenotypes that were markedly reduced upon pharmacologic inhibition of the HBP, suggesting activation of this pathway represents an adaptive mechanism to glutamine stress in KRAS/STK11-mutant LUAD cells.

Supporting this interpretation is the timing of the observed changes – during the early hours of glutamine deprivation, STK11 null cells transitioned from a hypermetabolic state to one exhibiting reduced mitochondrial respiration but increased or sustained SRC, indicating metabolic reprogramming rather than bioenergetic failure. Simultaneously, HBP activity was increased despite extracellular glutamine depletion. Together, these findings suggest that glutamine stress induces a coordinated redistribution of intracellular glutamine toward pathways that prioritize survival and metastatic fitness over oxidative metabolism.

The HBP is uniquely positioned to mediate this adaptive response as its end product, UDP-GlcNAc, supports both protein O-GlcNAcylation and surface glycosylation, processes known to regulate cell adhesion, migration, signaling, and immune interactions [34]. Shunting of nutrients through the HBP is relatively low under normal conditions, however upregulation is often observed in cancer, and is associated with more aggressive disease [40]. In our study, pharmacologic inhibition of the HBP with FR054 significantly reduced glutamine deprivation-induced cell detachment, impaired re-establishment after detachment, and suppressed spheroid invasion. These findings suggest that HBP activation is not merely a metabolic consequence of glutamine stress but a functional driver of the aggressive phenotype observed in KRAS/STK1 mutant LUAD cells.

Beyond regulating cell adhesion and glycosylation, enhanced HBP activity may couple metabolic adaptation to the transcriptional reprogramming induced by glutamine stress. We previously demonstrated that glutamine deprivation in KRAS/STK11-mutant LUAD activates multiple oncogenic pathways, including YAP1, NF-κB, TNF, HIF-1, chemokine, and focal adhesion signaling [14]. Our current findings suggest that increased HBP flux may act upstream of these transcriptional programs. O-GlcNAcylation is a nutrient-sensitive modification that regulates the activity of several oncogenic and tumor suppressive transcription factors, including YAP [41], NF-κB [18], p53 [42], and more. Thus, one possibility is that glutamine stress redirects intracellular glutamine toward the HBP, enabling glycosylation-dependent activation of transcriptional programs that promote metastatic phenotypes, yet this remains to be confirmed.

In summary, we demonstrate that glutamine deprivation induces metabolic rewiring in KRAS/STK11-mutant LUAD that redirects intracellular glutamine toward the HBP, promoting glycosylation-dependent processes that enhance metastatic behavior. These findings identify the HBP as a previously unrecognized adaptive response to glutamine stress and provide mechanistic support for the emerging hormetic model of glutamine metabolism [39]. Targeting both glutamine utilization and the adaptive HBP response may represent a more effective therapeutic strategy for patients with KRAS/STK11-mutant LUAD. Future work will determine whether the HBP functions upstream of nutrient-responsive transcriptional programs through which glutamine stress promotes metastatic features in KRAS/STK11-mutant LUAD.

## Supplementary Material

Supplemental Figure 1. STK11 loss drives enhanced glutamine-dependent mitochondrial respiration in KRAS-driven LUAD cells.

**(A)** Representative extracellular acidification rate (ECAR) traces of a Seahorse MST in NCI-H2009 KRAS^G12A^ and KRAS^G12A^ΔSTK11 cells treated with either 0 mM or 2 mM glutamine. Cells were seeded in respective glutamine deficient or replete media approximately 5 hours prior to and during the MST. **(B)** Representative extracellular acidification (ECAR) traces of a Seahorse MST in NCI-H441 KRAS^G12D^ and KRAS^G12D^ΔSTK11 cells treated with either 0 mM or 2 mM glutamine. Cells were seeded in respective glutamine deficient or replete media approximately 5 hours prior to and during the MST. **(C)** Quantification of H2009 KRAS^G12A^ and KRAS^G12A^ΔSTK11 secondary MST parameters (ATP-linked respiration, proton (H+) leak and coupling efficiency) in 0 mM and 2 mM glutamine conditions (n = 3; two-way ANOVA with Tukey’s multiple comparisons test). **(D)** Quantification of H441 KRAS^G12D^ and KRAS^G12D^ΔSTK11 secondary MST parameters (ATP-linked respiration, proton (H+) leak and coupling efficiency) in 0 mM and 2 mM glutamine conditions. **(E)** Representative traces of a Seahorse MST in NCI-H2009 KRAS^G12A^ and KRAS^G12A^ΔSTK11 cells treated with either 2 mM glutamine or 0 mM glutamine. Cells were seeded in either 2 mM or 0 mM glutamine approximately 5 hours prior to the MST, and 3-hour glutamine deprivation samples were swapped to 0 mM glutamine deficient media at 2 hours post-seeding. Oligo = oligomycin, R/A = rotenone/antimycin. * p < 0.05, ** p < 0.01, *** p < 0.001, **** p <0.0001, standard deviation (SD) shown.

Supplemental Figure 2. Top 50 unlabeled metabolites from modified glutamine pulse-chase.

Heat map of top 50 unlabeled metabolites by ANOVA p value in NCI-H2009 KRAS^G12A^ and KRAS^G12A^ΔSTK11 cells following a modified pulse-chase experiment in which cells were pulsed with mM ^13^C^15^N-glutamine for 4 hours which was then chased following exogenous glutamine deprivation for either 0 (i.e. immediately post-pulse), 6 or 24 hours. Analysis was performed via UHPLC-MS/MS at the University of Colorado Anschutz Metabolomics Core (n = 3).

Supplemental Figure 3. HBP activity induced upon glutamine deprivation promotes detachment and survival in KRAS/STK11-mutant LUAD cells.

Quantification of **(A)** NCI-H2009 KRAS^G12A^ and KRAS^G12A^ΔSTK11 and **(B)** NCI-H441 KRAS^G12V^ and KRAS^G12V^ΔSTK11 cells subjected to 24 hr of culture in 2 mM glutamine and 0 μM (i.e. DMSO vehicle) or 50 μM FR054, as indicated. Quantification was performed using Trypan Blue stain and analysis was done on Trypan Blue exclusive (i.e. live) versus inclusive (i.e. dead) cells (n = 3; one-way ANOVA with Tukey’s multiple comparisons test).

Supplemental Figure 4. FR054 diminishes invasive blebbing phenotype of H441 KRAS/STK11-mutant LUAD spheroids.

Representative images of NCI-H441 KRAS^G12V^ and KRAS^G12V^ΔSTK11 spheroid invasion at 0 and 72 hours following culture in **(A)** glutamine-replete (i.e. 2 mM) and **(B)** glutamine-deficient (i.e. 0 mM) conditions with concurrent 0 μM (i.e. DMSO vehicle) or 250 μM FR054 treatment, as indicated.

This is a list of supplementary files associated with this preprint. Click to download.

- SupplementalFigure1.tif
- SupplementalFigure2.tif
- SupplementalFigure3.tif
- SupplementalFigure4.tif
- Compiledrawblots.pdf
- H2009WT0mMGln.mp4
- H441STK11null2mMGln.mp4
- H441STK11null0mMGln.mp4
- H2009STK11null2mMGln.mp4
- H441WT2mMGln.mp4
- H2009STK11null0mMGln.mp4
- H441WT0mMGln.mp4
- H2009WT2mMGln.mp4

## Figures and Tables

**Figure 7.**
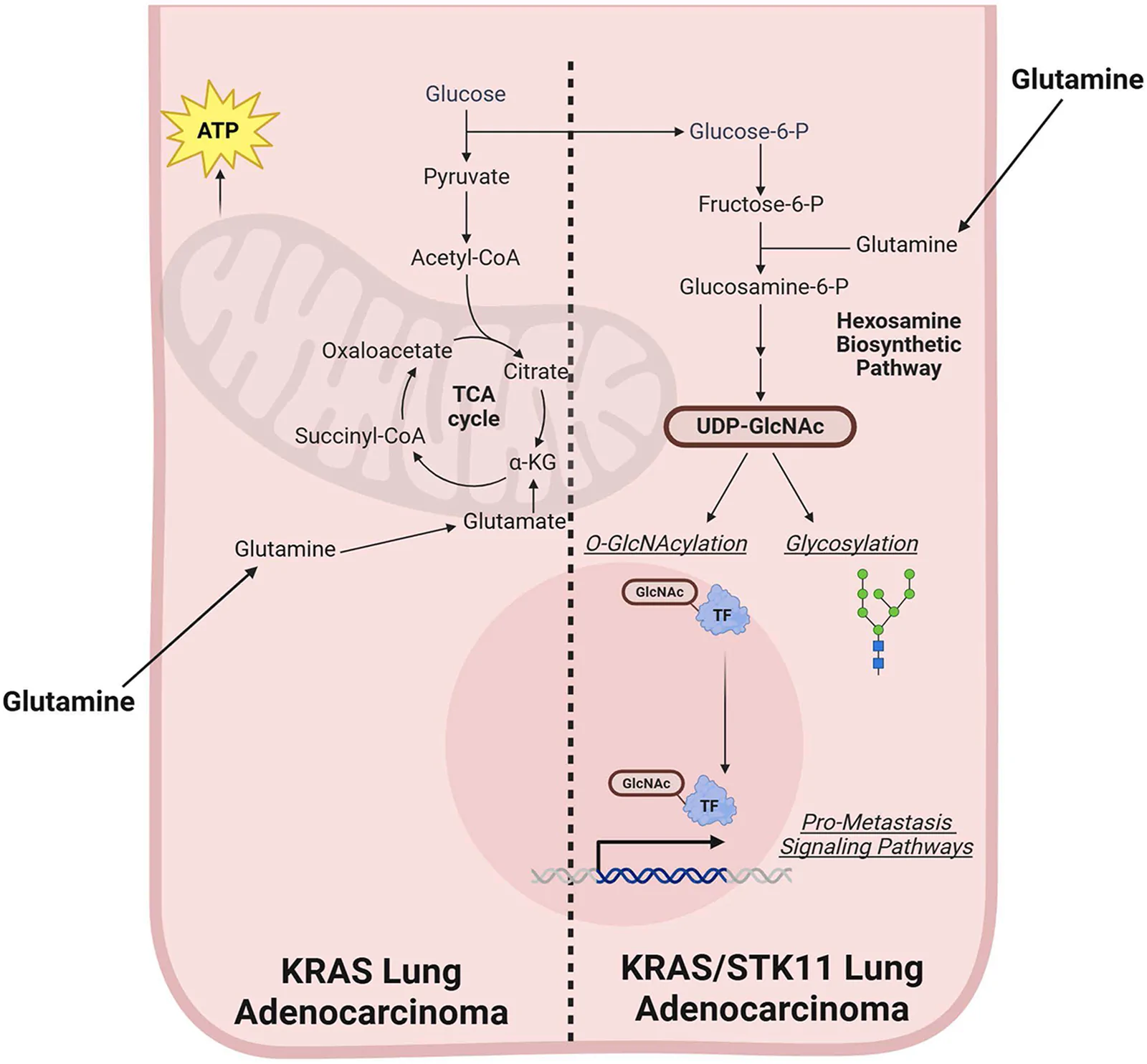
Working model. While KRAS lung adenocarcinoma (LUAD) primarily rewires glutamine metabolism to drive energy production via TCA flux and mitochondrial respiration, glutamine-deficient conditions such as the tumor microenvironment or glutaminase inhibition trigger a protective shunt of glutamine from the TCA to the hexosamine biosynthetic pathway (HBP) in KRAS/STK11-mutant LUAD. This rewired metabolism favoring HBP signaling rather than energetic production drives pro-metastatic signaling pathways that enable the KRAS/STK11-mutant LUAD cell to evade death by nutrient stress and, instead, disseminate to a distant location. As such, we propose that this pathway should be heavily considered when involving glutaminase inhibition as a treatment strategy for KRAS/STK11-mutant LUAD patients given that such an approach could promote more aggressive, metastatic disease.

## References

[1] Kratzer TB, Bandi P, Freedman ND, Smith RA, Travis WD, Jemal A, Siegel RL. Lung cancer statistics, 2023. Cancer. 2024;130(8):1330–48. 38279776 10.1002/cncr.35128

[2] Gleeson FC, Kipp BR, Levy MJ, Voss JS, Campion MB, Minot DM, Somatic STK11 and concomitant STK11/KRAS mutational frequency in stage IV lung adenocarcinoma adrenal metastases. J Thorac Oncol. 2015;10(3):531–4. 25695224 10.1097/JTO.0000000000000391

[3] Skoulidis F, Goldberg ME, Greenawalt DM, Hellmann MD, Awad MM, Gainor JF, STK11/LKB1 Mutations and PD-1 Inhibitor Resistance in KRAS-Mutant Lung Adenocarcinoma. Cancer Discov. 2018;8(7):822–35. 29773717 10.1158/2159-8290.CD-18-0099 PMC6030433

[4] Hezel AF, Bardeesy N. LKB1; linking cell structure and tumor suppression. Oncogene. 2008;27(55):6908–19. 19029933 10.1038/onc.2008.342

[5] Galan-Cobo A, Sitthideatphaiboon P, Qu X, Poteete A, Pisegna MA, Tong P, LKB1 and KEAP1/NRF2 Pathways Cooperatively Promote Metabolic Reprogramming with Enhanced Glutamine Dependence in KRAS-Mutant Lung Adenocarcinoma. Cancer Res. 2019;79(13):3251–67. 31040157 10.1158/0008-5472.CAN-18-3527 PMC6606351

[6] Zhang Y, Meng Q, Sun Q, Xu ZX, Zhou H, Wang Y. LKB1 deficiency-induced metabolic reprogramming in tumorigenesis and non-neoplastic diseases. Mol Metab. 2021;44:101131. 33278637 10.1016/j.molmet.2020.101131 PMC7753952

[7] Faubert B, Vincent EE, Griss T, Samborska B, Izreig S, Svensson RU, Loss of the tumor suppressor LKB1 promotes metabolic reprogramming of cancer cells via HIF-1α. Proc Natl Acad Sci U S A. 2014;111(7):2554–9. 24550282 10.1073/pnas.1312570111 PMC3932920

[8] Pan M, Reid MA, Lowman XH, Kulkarni RP, Tran TQ, Liu X, Regional glutamine deficiency in tumours promotes dedifferentiation through inhibition of histone demethylation. Nat Cell Biol. 2016;18(10):1090–101. 27617932 10.1038/ncb3410 PMC5536113

[9] Hojfeldt JW, Helin K. Regional tumour glutamine supply affects chromatin and cell identity. Nat Cell Biol. 2016;18(10):1027–9. 27684506 10.1038/ncb3414

[10] Recouvreux MV, Moldenhauer MR, Galenkamp KMO, Jung M, James B, Zhang Y, Glutamine depletion regulates Slug to promote EMT and metastasis in pancreatic cancer. J Exp Med. 2020;217(9). 10.1084/jem.20200388 PMC7478719 32510550

[11] Lee SW, Zhang Y, Jung M, Cruz N, Alas B, Commisso C. EGFR-Pak Signaling Selectively Regulates Glutamine Deprivation-Induced Macropinocytosis. Dev Cell. 2019;50(3):381–92 e5. 31257175 10.1016/j.devcel.2019.05.043 PMC6684838

[12] Commisso C, Davidson SM, Soydaner-Azeloglu RG, Parker SJ, Kamphorst JJ, Hackett S, Macropinocytosis of protein is an amino acid supply route in Ras-transformed cells. Nature. 2013;497(7451):633–7. 23665962 10.1038/nature12138 PMC3810415

[13] Kamphorst JJ, Nofal M, Commisso C, Hackett SR, Lu W, Grabocka E, Human pancreatic cancer tumors are nutrient poor and tumor cells actively scavenge extracellular protein. Cancer Res. 2015;75(3):544–53. 25644265 10.1158/0008-5472.CAN-14-2211 PMC4316379

[14] Lenahan SM, Sarausky HM, Deming P, Seward DJ. STK11 loss leads to YAP1-mediated transcriptional activation in human KRAS-driven lung adenocarcinoma cell lines. Cancer Gene Ther. 2024;31(1):1–8. 37968341 10.1038/s41417-023-00687-y PMC10794139

[15] Kim J, Lee HM, Cai F, Ko B, Yang C, Lieu EL, The hexosamine biosynthesis pathway is a targetable liability in KRAS/LKB1 mutant lung cancer. Nat Metab. 2020;2(12):1401–12. 33257855 10.1038/s42255-020-00316-0 PMC7744327

[16] Marshall S, Bacote V, Traxinger RR. Discovery of a metabolic pathway mediating glucose-induced desensitization of the glucose transport system. Role of hexosamine biosynthesis in the induction of insulin resistance. J Biol Chem. 1991;266(8):4706–12. 2002019

[17] Lam C, Low JY, Tran PT, Wang H. The hexosamine biosynthetic pathway and cancer: Current knowledge and future therapeutic strategies. Cancer Lett. 2021;503:11–8. 33484754 10.1016/j.canlet.2021.01.010

[18] Ma Z, Chalkley RJ, Vosseller K. Hyper-O-GlcNAcylation activates nuclear factor κ-light-chain-enhancer of activated B cells (NF-κB) signaling through interplay with phosphorylation and acetylation. J Biol Chem. 2017;292(22):9150–63. 28416608 10.1074/jbc.M116.766568 PMC5454098

[19] Freund P, Kerenyi MA, Hager M, Wagner T, Wingelhofer B, Pham HTT, O-GlcNAcylation of STAT5 controls tyrosine phosphorylation and oncogenic transcription in STAT5-dependent malignancies. Leukemia. 2017;31(10):2132–42. 28074064 10.1038/leu.2017.4 PMC5629373

[20] Das S, Bailey SK, Metge BJ, Hanna A, Hinshaw DC, Mota M, O-GlcNAcylation of GLI transcription factors in hyperglycemic conditions augments Hedgehog activity. Lab Invest. 2019;99(2):260–70. 30420690 10.1038/s41374-018-0122-8 PMC6857801

[21] Gu X, Ma Y, Liu Y, Wan Q. Measurement of mitochondrial respiration in adherent cells by Seahorse XF96 Cell Mito Stress Test. STAR Protoc. 2021;2(1):100245. 33458707 10.1016/j.xpro.2020.100245 PMC7797920

[22] Gebeyehu A, Surapaneni SK, Huang J, Mondal A, Wang VZ, Haruna NF, Polysaccharide hydrogel based 3D printed tumor models for chemotherapeutic drug screening. Sci Rep. 2021;11(1):372. 33431915 10.1038/s41598-020-79325-8 PMC7801509

[23] Foty R. A simple hanging drop cell culture protocol for generation of 3D spheroids. J Vis Exp. 2011(51). 10.3791/2720 PMC3197119 21587162

[24] Caiola E, Falcetta F, Giordano S, Marabese M, Garassino MC, Broggini M, Co-occurring KRAS mutation/LKB1 loss in non-small cell lung cancer cells results in enhanced metabolic activity susceptible to caloric restriction: an in vitro integrated multilevel approach. J Exp Clin Cancer Res. 2018;37(1):302. 30514331 10.1186/s13046-018-0954-5 PMC6280460

[25] Roberts E, Simonsen DG, Tanaka KK, Tanaka T. Free amino acids in growing and regressing ascites cell tumors: host resistance and chemical agents. Cancer Res. 1956;16(10 Part 1):970–8. 13374707

[26] Rivera S, Azcón-Bieto J, López-Soriano FJ, Miralpeix M, Argilés JM. Amino acid metabolism in tumour-bearing mice. Biochem J. 1988;249(2):443–9. 3342022 10.1042/bj2490443 PMC1148723

[27] Márquez J, Sánchez-Jiménez F, Medina MA, Quesada AR, Núñez de Castro I. Nitrogen metabolism in tumor bearing mice. Arch Biochem Biophys. 1989;268(2):667–75. 2913952 10.1016/0003-9861(89)90335-4

[28] Fardini Y, Dehennaut V, Lefebvre T, Issad T. O-GlcNAcylation: A New Cancer Hallmark? Front Endocrinol (Lausanne). 2013;4:99. 23964270 10.3389/fendo.2013.00099 PMC3740238

[29] Dobie C, Skropeta D. Insights into the role of sialylation in cancer progression and metastasis. Br J Cancer. 2021;124(1):76–90. 33144696 10.1038/s41416-020-01126-7 PMC7782833

[30] Magnelli P, Bielik A, Guthrie E. Identification and characterization of protein glycosylation using specific endo- and exoglycosidases. Methods Mol Biol. 2012;801:189–211. 21987255 10.1007/978-1-61779-352-3_13

[31] Ricciardiello F, Votta G, Palorini R, Raccagni I, Brunelli L, Paiotta A, Inhibition of the Hexosamine Biosynthetic Pathway by targeting PGM3 causes breast cancer growth arrest and apoptosis. Cell Death Dis. 2018;9(3):377. 29515119 10.1038/s41419-018-0405-4 PMC5841296

[32] Lee H, Cai F, Kelekar N, Velupally NK, Kim J. Targeting PGM3 as a Novel Therapeutic Strategy in KRAS/LKB1 Co-Mutant Lung Cancer. Cells. 2022;11(1). 10.3390/cells11010176 PMC8750012 35011738

[33] Fares J, Fares MY, Khachfe HH, Salhab HA, Fares Y. Molecular principles of metastasis: a hallmark of cancer revisited. Signal Transduct Target Ther. 2020;5(1):28. 32296047 10.1038/s41392-020-0134-x PMC7067809

[34] Chiaradonna F, Ricciardiello F, Palorini R. The Nutrient-Sensing Hexosamine Biosynthetic Pathway as the Hub of Cancer Metabolic Rewiring. Cells. 2018;7(6). 10.3390/cells7060053 PMC6025041 29865240

[35] Kumar R, Saneja A, Panda AK. An Annexin V-FITC-Propidium Iodide-Based Method for Detecting Apoptosis in a Non-Small Cell Lung Cancer Cell Line. Methods Mol Biol. 2021;2279:213–23. 33683697 10.1007/978-1-0716-1278-1_17

[36] Wu JS, Jiang J, Chen BJ, Wang K, Tang YL, Liang XH. Plasticity of cancer cell invasion: Patterns and mechanisms. Transl Oncol. 2021;14(1):100899. 33080522 10.1016/j.tranon.2020.100899 PMC7573380

[37] Pandya P, Orgaz JL, Sanz-Moreno V. Modes of invasion during tumour dissemination. Mol Oncol. 2017;11(1):5–27. 28085224 10.1002/1878-0261.12019 PMC5423224

[38] Dillekås H, Rogers MS, Straume O. Are 90% of deaths from cancer caused by metastases? Cancer Med. 2019;8(12):5574–6. 31397113 10.1002/cam4.2474 PMC6745820

[39] Grenier SF, Commisso C. A hormetic response model for glutamine stress in cancer. Trends Cancer. 2025;11(3):196–203. 39681506 10.1016/j.trecan.2024.11.008 PMC11903170

[40] Abdel Rahman AM, Ryczko M, Pawling J, Dennis JW. Probing the hexosamine biosynthetic pathway in human tumor cells by multitargeted tandem mass spectrometry. ACS Chem Biol. 2013;8(9):2053–62. 23875632 10.1021/cb4004173

[41] Peng C, Zhu Y, Zhang W, Liao Q, Chen Y, Zhao X, Regulation of the Hippo-YAP Pathway by Glucose Sensor O-GlcNAcylation. Mol Cell. 2017;68(3):591–604.e5. 29100056 10.1016/j.molcel.2017.10.010

[42] Yang WH, Kim JE, Nam HW, Ju JW, Kim HS, Kim YS, Cho JW. Modification of p53 with O-linked N-acetylglucosamine regulates p53 activity and stability. Nat Cell Biol. 2006;8(10):1074–83. 16964247 10.1038/ncb1470

